# Supplementary material for: Discovery and application of insertion-deletion (INDEL) polymorphisms for QTL mapping of early life-history traits in Atlantic salmon
Source: BMC Genomics. 2010 Mar 8;11:156. doi: 10.1186/1471-2164-11-156 (PMC2838853; doi:10.1186/1471-2164-11-156)
Supplement: Additional file 2 — Information on developed 76 locus single-run INDEL panel in Atlantic salmon. Information on fluorescence labeling, primer concentrations, PCR pooling and links to alignments, INDEL motifs and GENESCAN (Burge and Karlin 1997) predictions of genes/exons are available in html format. [file 1471-2164-11-156-S2.ZIP › Additionalfile2/table4955.html]

Contig Alignment

```
Base	A B C D E F G H I J K L M N O   
1	. G . . . . . . . . . . . . .    
2	G G G . . . . . . . . . . . .    
3	G G G . . . . . . . . . . . .    
4	G G G . . . . . . . . . . . .    
5	G G G . . . . . . . . . . . .    
6	G G G . . . . . . . . . . . .    
7	A A A . . . . . . . . . . . .    
8	G G G . . . . . . . . . . . .    
9	A A A . . . . . . . . . . . .    
10	G G G . . . . . . . . . . . .    
11	T T T . . . . . . . . . . . .    
12	C C C . . . . . . . . . . . .    
13	A A A . . . . . . . . . . . .    
14	C C C . . . . . . . . . . . .    
15	A A A . . . . . . . . . . . .    
16	G G G . . . . . . . . . . . .    
17	T T T . . . . . . . . . . . .    
18	G G G . . . . . . . . . . . .    
19	T T T . . . . . . . . . . . .    
20	G G G . . . . . . . . . . . .    
21	T T T . . . . . . . . . . . .    
22	A A A . . . . . . . . . . . .    
23	C C C . . . . . . . . . . . .    
24	T T T . . . . . . . . . . . .    
25	T T T . . . . . . . . . . . .    
26	A A A . . . . . . . . . . . .    
27	T T T . . . . . . . . . . . .    
28	T T T . . . . . . . . . . . .    
29	G G G . . . . . . . . . . . .    
30	T T T . . . . . . . . . . . .    
31	T T T . . . . . . . . . . . .    
32	T T T . . . . . . . . . . . .    
33	G G G . . . . . . . . . . . .    
34	T T T . . . . . . . . . . . .    
35	G G G . . . . . . . . . . . .    
36	G G G . . . . . . . . . . . .    
37	T T T . . . . . . . . . . . .    
38	C C C . . . . . . . . . . . .    
39	T T T . . . . . . . . . . . .    
40	G G G . . . . . . . . . . . .    
41	T T T . . . . . . . . . . . .    
42	G G G . . . . . . . . . . . .    
43	G G G . . . . . . . . . . . .    
44	C C C . . . . . . . . . . . .    
45	T T T . . . . . . . . . . . .    
46	A A A . . . . . . . . . . . .    
47	G G G . . . . . . . . . . . .    
48	C C C . . . . . . . . . . . .    
49	T T T . . . . . . . . . . . .    
50	A A A . . . . . . . . . . . .    
51	G G G . . . . . . . . . . . .    
52	C C C . . . . . . . . . . . .    
53	T T T . . . . . . . . . . . .    
54	A A A . . . . . . . . . . . .    
55	G G G . . . . . . . . . . . .    
56	A G A . . . . . . . . . . . .    
57	C C C . . . . . . . . . . . .    
58	A A A . . . . . . . . . . . .    
59	T T T . . . . . . . . . . . .    
60	A A A . . . . . . . . . . . .    
61	T T T . . . . . . . . . . . .    
62	T T T . . . . . . . . . . . .    
63	G G G G . . . . . . . . . . .    
64	G G G G . . . . . . . . . . .    
65	C C C C . . . . . . . . . . .    
66	T T T T . . . . . . . . . . .    
67	A A A A . . . . . . . . . . .    
68	T T T T . . . . . . . . . . .    
69	A A A A . . . . . . . . . . .    
70	T T T T . . . . . . . . . . .    
71	T T T T . . . . . . . . . . .    
72	T T T T . . . . . . . . . . .    
73	G G G G . . . . . . . . . . .    
74	A A A A . . . . . . . . . . .    
75	C C C C . . . . . . . . . . .    
76	A A A A . . . . . . . . . . .    
77	A A A A . . . . . . . . . . .    
78	A A A A . . . . . . . . . . .    
79	A A A A . . . . . . . . . . .    
80	C C C C . . . . . . . . . . .    
81	A A A A . . . . . . . . . . .    
82	C C C C . . . . . . . . . . .    
83	T T T T . . . . . . . . . . .    
84	A A A A . . . . . . . . . . .    
85	C C C C . . . . . . . . . . .    
86	C C C C . . . . . . . . . . .    
87	G G G G . . . . . . . . . . .    
88	T T T T . . . . . . . . . . .    
89	A A A A . . . . . . . . . . .    
90	A A A A . . . . . . . . . . .    
91	C C C C . . . . . . . . . . .    
92	G G G G . . . . . . . . . . .    
93	T T T T . . . . . . . . . . .    
94	T T T T . . . . . . . . . . .    
95	A A A A . . . . . . . . . . .    
96	A A A A . . . . . . . . . . .    
97	C C C C . . . . . . . . . . .    
98	G G G G . . . . . . . . . . .    
99	C C C C . . . . . . . . . . .    
100	A A A A . . . . . . . . . . .    
101	A A A A . . . . . . . . . . .    
102	A A A A . . . . . . . . . . .    
103	G G G G . . . . . . . . . . .    
104	G G G G . . . . . . . . . . .    
105	A A A A . . . . . . . . . . .    
106	G G G G . . . . . . . . . . .    
107	G G G G . . . . . . . . . . .    
108	G G G G . . . . . . . . . . .    
109	A A A A . . . . . . . . . . .    
110	G G G G . . . . . . . . . . .    
111	G G G G . . . . . . . . . . .    
112	A A A A . . . . . . . . . . .    
113	A A A A . . . . . . . . . . .    
114	T T T T . . . . . . . . . . .    
115	C C C C . . . . . . . . . . .    
116	C C C C . . . . . . . . . . .    
117	A A A A . . . . . . . . . . .    
118	A A A A . . . . . . . . . . .    
119	G G G G . . . . . . . . . . .    
120	T T T T . . . . . . . . . . .    
121	G G G G . . . . . . . . . . .    
122	A A A A . . . . . . . . . . .    
123	G G G G . . . . . . . . . . .    
124	C C C C . . . . . . . . . . .    
125	A A A A . . . . . . . . . . .    
126	C C C C . . . . . . . . . . .    
127	T T T T . . . . . . . . . . .    
128	G G G G . . . . . . . . . . .    
129	A A A A . . . . . . . . . . .    
130	A A A A . . . . . . . . . . .    
131	T T T T . . . . . . . . . . .    
132	C C C C . . . . . . . . . . .    
133	C C C C . . . . . . . . . . .    
134	T T T T . . . . . . . . . . .    
135	G G G G . . . . . . . . . . .    
136	G G G G . . . . . . . . . . .    
137	A A A A . . . . . . . . . . .    
138	T T T T . . . . . . . . . . .    
139	A A A A . . . . . . . . . . .    
140	G G G G . . . . . . . . . . .    
141	A A A A . . . . . . . . . . .    
142	A A A A . . . . . . . . . . .    
143	G G G G . . . . . . . . . . .    
144	T T T T . . . . . . . . . . .    
145	C C C C . . . . . . . . . . .    
146	A A A A . . . . . . . . . . .    
147	C C C C . . . . . . . . . . .    
148	G G G G . . . . . . . . . . .    
149	T T T T . . . . . . . . . . .    
150	C C C C . . . . . . . . . . .    
151	G G G G . . . . . . . . . . .    
152	C C C C . . . . . . . . . . .    
153	T T T T . . . . . . . . . . .    
154	A A A A . . . . . . . . . . .    
155	A A A A . . . . . . . . . . .    
156	A A A A . . . . . . . . . . .    
157	A A A A . . . . . . . . . . .    
158	G G G G . . . . . . . . . . .    
159	A A A A . . . . . . . . . . .    
160	A A A A . . . . . . . . . . .    
161	G G G G . . . . . . . . . . .    
162	A A A A . . . . . . . . . . .    
163	A A A A . . . . . . . . . . .    
164	A A A A . . . . . . . . . . .    
165	T T T T . . . . . . . . . . .    
166	A A A A . . . . . . . . . . .    
167	T T T T . . . . . . . . . . .    
168	G G G G . . . . . . . . . . .    
169	T T T T . . . . . . . . . . .    
170	T T T T . . . . . . . . . . .    
171	T T T T . . . . . . . . . . .    
172	C C C C . . . . . . . . . . .    
173	T T T T . . . . . . . . . . .    
174	A A A A . . . . . . . . . . .    
175	T T T T . . . . . . . . . . .    
176	C C C C . . . . . . . . . . .    
177	C C C C . . . . . . . . . . .    
178	A A A A . . . . . . . . . . .    
179	A A A A . . . . . . . . . . .    
180	A A A A . . . . . . . . . . .    
181	C C C C . . . . . . . . . . .    
182	C C C C . . . . . . . . . . .    
183	A A A A . . . . . . . . . . .    
184	G G G G . . . . . . . . . . .    
185	T T T T . . . . . . . . . . .    
186	A A A A . . . . . . . . . . .    
187	G G G G . . . . . . . . . . .    
188	A A A A . . . . . . . . . . .    
189	A A A A . . . . . . . . . . .    
190	A A A A . . . . . . . . . . .    
191	A A A A . . . . . . . . . . .    
192	C C C C . . . . . . . . . . .    
193	T T T T . . . . . . . . . . .    
194	G G G G . . . . . . . . . . .    
195	A A A A . . . . . . . . . . .    
196	C C C C . . . . . . . . . . .    
197	T T T T . . . . . . . . . . .    
198	G G G G . . . . . . . . . . .    
199	G G G G . . . . . . . . . . .    
200	T T T T . . . . . . . . . . .    
201	T T T T . . . . . . . . . . .    
202	G G G G . . . . . . . . . . .    
203	G G G G . . . . . . . . . . .    
204	C C C C . . . . . . . . . . .    
205	C C C C . . . . . . . . . . .    
206	A A A A . . . . . . . . . . .    
207	G G G G . . . . . . . . . . .    
208	C C C C . . . . . . . . . . .    
209	T T T T . . . . . . . . . . .    
210	C C C C . . . . . . . . . . .    
211	A A A A . . . . . . . . . . .    
212	T T T T . . . . . . . . . . .    
213	A A A A . . . . . . . . . . .    
214	T T T T . . . . . . . . . . .    
215	A A A A . . . . . . . . . . .    
216	A A A A . . . . . . . . . . .    
217	T T T T . . . . . . . . . . .    
218	C C C C . . . . . . . . . . .    
219	G G G G . . . . . . . . . . .    
220	T T T T . . . . . . . . . . .    
221	T T T T . . . . . . . . . . .    
222	G G G G . . . . . . . . . . .    
223	C C C C . . . . . . . . . . .    
224	C C C C . . . . . . . . . . .    
225	T T T T . . . . . . . . . . .    
226	A A A A . . . . . . . . . . .    
227	G G G G . . . . . . . . . . .    
228	G G G G . . . . . . . . . . .    
229	A A A A . . . . . . . . . . .    
230	G G G G . . . . . . . . . . .    
231	T T T T . . . . . . . . . . .    
232	T T T T . . . . . . . . . . .    
233	T T T T . . . . . . . . . . .    
234	T T T T . . . . . . . . . . .    
235	C C C C . . . . . . . . . . .    
236	T T T T . . . . . . . . . . .    
237	G G G G . . . . . . . . . . .    
238	T T T T . . . . . . . . . . .    
239	G G G G . . . . . . . . . . .    
240	A A A A . . . . . . . . . . .    
241	C C C C . . . . . . . . . . .    
242	A A A A . . . . . . . . . . .    
243	A A A A . . . . . . . . . . .    
244	G G G G . . . . . . . . . . .    
245	G G G G . . . . . . . . . . .    
246	G G G G . . . . . . . . . . .    
247	A A A A . . . . . . . . . . .    
248	T T T T . . . . . . . . . . .    
249	A A A A . . . . . . . . . . .    
250	A A A A . . . . . . . . . . .    
251	T T T T . . . . . . . . . . .    
252	G G G G . . . . . . . . . . .    
253	T T T T . . . . . . . . . . .    
254	C C C C . . . . . . . . . . .    
255	C C C C . . . . . . . . . . .    
256	C C C C . . . . . . . . . . .    
257	A A A A . . . . . . . . . . .    
258	C C C C . . . . . . . . . . .    
259	C C C C . . . . . . . . . . .    
260	A A A A . . . . . . . . . . .    
261	C C C C . . . . . . . . . . .    
262	T T T T . . . . . . . . . . .    
263	G G G G . . . . . . . . . . .    
264	A A A A . . . . . . . . . . .    
265	A A A A . A . . . . . . . . .    
266	G G G G . G . . . . . . . . .    
267	A A A A . A . . . . . . . . .    
268	T T T T . T . . . . . . . . .    
269	G G G G . G . . . . . . . . .    
270	G G G G . G . . . . . . . . .    
271	G G G G . G . . . . . . . . .    
272	A A A A . A . . . . . . . . .    
273	G G G G . G . . . . . . . . .    
274	G G G G . G . . . . . . . . .    
275	C C C C . C . . . . . . . . .    
276	A A A A . A . . . . . . . . .    
277	G G G G . G . . . . . . . . .    
278	T T T T . T . . . . . . . . .    
279	G G G G . G . . . . . . . . .    
280	G G G G . G . . . . . . . . .    
281	A A A A . A . . . . . . . . .    
282	T T T T . T . . . . . . . . .    
283	C C C C . C . . . . . . . . .    
284	C C C C . C . . . . . . . . .    
285	A A A A . A . . . . . . . . .    
286	A A A A . A . . . . . . . . .    
287	G G G G . G . . . . . . . . .    
288	G G G G . G . . . . . . . . .    
289	T T T T . T . . . . . . . . .    
290	G G G G . G . . . . . . . . .    
291	A A A A . A . . . . . . . . .    
292	A A A A . A . . . . . . . . .    
293	A A A A . A . . . . . . . . .    
294	G G G G . G . . . . . . . . .    
295	G G G G . G . . . . . . . . .    
296	T T T T . T . . . . . . . . .    
297	G G G G . G . . . . . . . . .    
298	C C C C . C . . . . . . . . .    
299	T T T T . T . . . . . . . . .    
300	T T T T . T . . . . . . . . .    
301	G G G G . G . . . . . . . . .    
302	G G G G . G . . . . . . . . .    
303	C C C C . C . . . . . . . . .    
304	C C C C . C . . . . . . . . .    
305	A A A A . A . . . . . . . . .    
306	T T T T . T . . . . . . . . .    
307	T T T T . T . . . . . . . . .    
308	T T T T . T . . . . . . . . .    
309	C C C C . C . . . . . . . . .    
310	C C C C . C . . . . . . . . .    
311	T T T T . T . . . . . . . . .    
312	G G G G . G . . . . . . . . .    
313	G G G G . G . . . . . . . . .    
314	T T T T . T . . . . . . . . .    
315	T T T T . T . . . . . . . . .    
316	C C C C . C . . . . . . . . .    
317	G G G G . G G . . . . . . . .    
318	G G G G . G G . . . . . . . .    
319	G G G G . G G . . . . . . . .    
320	A A A A . A A . . . . . . . .    
321	G G G G . G G . . . . . . . .    
322	C C C C . C C . . . . . . . .    
323	T T T T . T T . . . . . . . .    
324	G G G G . G G . . . . . . . .    
325	G G G G . G G . . . . . . . .    
326	A A A A . A A A . . . . . . .    
327	G G G G . G G G . . . . . . .    
328	G G G G . G G G . . . . . . .    
329	G G G G . G G G . . . . . . .    
330	G G G G . G G G . . . . . . .    
331	A A A A . A A A . . . . . . .    
332	T T T T . T T T . . . . . . .    
333	T T T T . T T T . . . . . . .    
334	C C C C . C C C . . . . . . .    
335	C C C C . C C C . . . . . . .    
336	G G G G . G G G . . . . . . .    
337	G G G G . G G G . . . . . . .    
338	C C C C . C C C . . . . . . .    
339	A A A A . A A A . . . . . . .    
340	G G G G . G G G . . . . . . .    
341	T T T T . T T T . . . . . . .    
342	G G G G . G G G . . . . . . .    
343	A A A A . A A A . . . . . . .    
344	G G G G . G G G . . . . . . .    
345	G G G G . G G G . . . . . . .    
346	G G G G . G G G . . . . . . .    
347	G G G G . G G G . . . . . . .    
348	C C C C . C C C . . . . . . .    
349	A A A A . A A A . . . . . . .    
350	A A A A . A A A . . . . . . .    
351	G G G G . G G G . . . . . . .    
352	A A A A . A A A . . . . . . .    
353	A A A A . A A A . . . . . . .    
354	G G G G . G G G . . . . . . .    
355	A A A A . A A A . . . . . . .    
356	T T T T . T T T . . . . . . .    
357	C C C C . C C C . . . . . . .    
358	A A A A . A A A . . . . . . .    
359	C C C C . C C C . . . . . . .    
360	T T T T . T T T . . . . . . .    
361	C C C C . C C C . . . . . . .    
362	T T T T . T T T . . . . . . .    
363	G G G G . G G G . . . . . . .    
364	T T T T . T T T . . . . . . .    
365	T T T T . T T T . . . . . . .    
366	G G G G . G G G . . . . . . .    
367	C C C C . C C C . . . . . . .    
368	C C C C . C C C . . . . . . .    
369	C C C C . C C C . . . . . . .    
370	G G G G . G G G . . . . . . .    
371	A A A A . A A A . . . . . . .    
372	C C C C . C C C . . . . . . .    
373	T T T T . T T T . . . . . . .    
374	C C C C . C C C . . . . . . .    
375	A A A A . A A A . . . . . . .    
376	A A A A . A A A . . . . . . .    
377	A A A A . A A A . . . . . . .    
378	G G G G . G G G . . . . . . .    
379	G G G G . G G G . . . . . . .    
380	G G G G . G G G . . . . . . .    
381	A A A A . A A A . . . . . . .    
382	C C C C . C C C . . . . . . .    
383	C C C C . C C C . . . . . . .    
384	A A A A . A A A . . . . . . .    
385	G G G G . G G G . . . . . . .    
386	A A A A . A A A . . . . . . .    
387	A A A A . A A A . . . . . . .    
388	A A A A . A A A . . . . . . .    
389	A A A A . A A A . . . . . . .    
390	G G G G . G G G . . . . . . .    
391	C C C C . C C C . . . . . . .    
392	A A A A . A A A . . . . . . .    
393	A A A A . A A A . . . . . . .    
394	C C C C . C C C . . . . . . .    
395	C C C C . C C C . . . . . . .    
396	C C C C . C C C . . . . . . .    
397	C C C C . C C C . . . . . . .    
398	A A A A . A A A . . . . . . .    
399	T T T T . T T T . . . . . . .    
400	T T T T . T T T . . . . . . .    
401	T T T T . T T T . . . . . . .    
402	A A A A . A A A . . A . . . .    
403	T T T T . T T T . . T . . . .    
404	T T T T . T T T . . T . . . .    
405	T T T T . T T T . . T . . . .    
406	T T T T . T T T . . T . . . .    
407	C C C C . C C C . . C . . . .    
408	A A A A . A A A . . A . . . .    
409	C C C C . C C C . . C . . . .    
410	A A A A . A A A . . A . . . .    
411	A A A A . A A A . . A . . . .    
412	G G G G . G G G . . G . . . .    
413	G G G G . G G G . . G . . . .    
414	A A A A . A A A . . A . . . .    
415	G G G G . G G G . . G . . . .    
416	G G G G . G G G . . G . . . .    
417	A A A A . A A A . . A . . . .    
418	G G G G . G G G . . G . . . .    
419	C C C C . C C C . . C . . . .    
420	T T T T . T T T . . T . . . .    
421	C C C C . C C C . . C . . . .    
422	T T T T . T T T . . T . . . .    
423	C C C C . C C C . . C . . . .    
424	T T T T . T T T . . T . . . .    
425	C C C C . C C C . . C . . . .    
426	T T T T . T T T . . T . . . .    
427	A A A A . A A A . . A . . . .    
428	C C C C . C C C . . C . . . .    
429	T T T T . T T T . . T . . . .    
430	A A A A . A A A . . A . . . .    
431	T T T T . T T T . . T . . . .    
432	G G G G . G G G . G G . . . .    
433	A A A A . A A A . A A . . . .    
434	T T T T . T T T . T T . . . .    
435	G G G G . G G G . G G . . . .    
436	A A A A . A A A . A A . . . .    
437	G G G G . G G G . G G . . . .    
438	G G G G . G G G . G G . . . .    
439	A A A A . A A A . A A . . . .    
440	T T T T . T T T . T T . . . .    
441	G G G G . G G G . G G . . . .    
442	G G G G . G G G . G G . . . .    
443	G G G G . G G G . G G . . . .    
444	G G G G . G G G . G G . . . .    
445	A A A A . A A A . A A . . . .    
446	C C C C . C C C . C C . . . .    
447	C C C C . C C C . C C . . . .    
448	T T T T . T T T . T T . . . .    
449	G G G G . G G G . G G . . . .    
450	G G G G . G G G . G G . . . .    
451	C C C C . C C C . C C . . . .    
452	T T T T . T T T . T T . . . .    
453	C C C C . C C C . C C . . . .    
454	A A A A . A A A . A A . . . .    
455	T T T T . T T T . T T . . . .    
456	G G G G . G G G . G G . . . .    
457	A A A A . A A A . A A . . . .    
458	G G G G . G G G . G G . . . .    
459	T T T T . T T T . T T . . . .    
460	T T T T . T T T . T T . . . .    
461	C C C C . C C C . C C . . . .    
462	T T T T . T T T . T T . . . .    
463	A A A A . A A A . A A . . . .    
464	T T T T . T T T . T T . . . .    
465	G G G G . G G G . G G . . . .    
466	A A A A . A A A . A A . . A .    
467	A A A A . A A A . A A . . A .    
468	G G G G . G G G . G G . . G .    
469	A A A A . A A A . A A . . A .    
470	G G G G . G G G . G G . . G .    
471	A A A A . A A A . A A . A A .    
472	C C C C . C C C . C C . C C .    
473	T T T T . T T T . T T . T T .    
474	G G G G . G G G . G G . G G .    
475	T T T T . T T T . T T . T T .    
476	A A A A . A A A . A A . A A .    
477	G G G G . G G G . G G . G G .    
478	T T T T . T T T . T T . T T .    
479	G G G G . G G G . G G . G G .    
480	A A A A . A A A . A A . A A .    
481	C C C C . C C C . C C . C C .    
482	A A A A . A A A . A A . A A .    
483	A A A A . A A A . A A . A A .    
484	A A A A . A A A . A A . A A .    
485	G G G G . G G G . G G . G G .    
486	A A A A . A A A . A A . A A .    
487	A A A A . A A A . A A . A A .    
488	T T T T . T T T . T T . T T .    
489	G G G G . G G G . G G . G G .    
490	G G G G . G G G . G G . G G .    
491	C C C C . C C C . C C . C C .    
492	C C C C . C C C . C C . C C .    
493	G G G G . G G G . G G . G G .    
494	G G G G . G G G . G G . G G .    
495	A A A A . A A A . A A . A A .    
496	A A A A . A A A . A A . A A .    
497	G G G G . G G G . G G . G G .    
498	A A A A . A A A . A A . A A .    
499	G G G G . G G G . G G . G G .    
500	G G G G . G G G . G G . G G .    
501	T T T T T T T T . T T . T T .    
502	C C C C C C C C . C C . C C .    
503	C C C C C C C C . C C . C C .    
504	A A A A A A A A . A A . A A .    
505	A A A A A A A A . A A . A A .    
506	G G G G G G G G . G G . G G .    
507	C C C C C C C C . C C . C C .    
508	T T T T T T T T . T T . T T .    
509	G G G G G G G G . G G . G G .    
510	A A A A A A A A . A A . A A .    
511	A A A A A A A A . A A . A A .    
512	G G G G G G G G . G G . G G .    
513	A A A A A A A A . A A . A A .    
514	G G G G G G G G . G G . G G .    
515	G G G G G G G G . G G . G G .    
516	A A A A A A A A . A A . A A .    
517	T T T T T T T T . T T . T T .    
518	T T T T T T T T . T T . T T .    
519	C C C C C C C C . C C . C C .    
520	A A A A A A A A . A A . A A .    
521	G G G G G G G G . G G . G G .    
522	A A A A A A A A . A A . A A .    
523	A A A A A A A A . A A . A A .    
524	G G G G G G G G . G G . G G .    
525	A A A A A A A A . A A . A A .    
526	A A A A A A A A . A A . A A .    
527	C C C C C C C C . C C . C C .    
528	C C C C C C C C . C C . C C .    
529	T T T T T T T T . T T . T T .    
530	C C C C C C C C . C C . C C .    
531	A A A A A A A A . A A . A A .    
532	T T T T T T T T . T T . T T .    
533	A A A A A A A A . A A . A A .    
534	C C C C C C C C . C C . C C .    
535	C C C C C C C C . C C . C C .    
536	T T T T T T T T . T T . T T .    
537	C C C C C C C C . C C . C C .    
538	A A A A A A A A . A A . A A .    
539	G G G G G G G G . G G . G G .    
540	G G G G G G G G . G G . G G .    
541	G G G G G G G G . G G . G G .    
542	A A A A A A A A . A A . A A .    
543	A A A A A A A A . A A . A A .    
544	T T T T T T T T . T T . T T .    
545	T T T T T T T T . T T . T T .    
546	G G G G G G G G . G G . G G .    
547	T T T T T T T T . T T . T T .    
548	G G G G G G G G . G G . G G .    
549	A A A A A A A A . A A . A A .    
550	A A A A A A A A . A A . A A .    
551	G G G G G G G G . G G . G G .    
552	C C C C C C C C . C C . C C .    
553	T T T T T T T T . T T . T T .    
554	A A A A A A A A . A A . A A .    
555	A A A A A A A A . A A . A A .    
556	A A A A A A A A . A A A A A .    
557	C C C C C C C C . C C C C C .    
558	C C C C C C C C . C C C C C .    
559	A A A A A A A A . A A A C A .    
560	C C C C C C C C . C C C C C .    
561	C C C C C C C C . C C C C C .    
562	C C C C C C C C . C C C C C .    
563	C C C C C C C C . C C C C C .    
564	C C C C C C C C . C C C C C .    
565	G G G G G G G G . G G G G G .    
566	G G G G G G G G . G G G G G .    
567	A A A A A A A A . A A A A A .    
568	T T T T T T T T . T T T T T .    
569	C C C C C C C C . C C C C C .    
570	C C C C C C C C . C C C C C .    
571	A A A A A A A A . A A A A A .    
572	T T T T T T T T . T T T T T .    
573	G G G G G G G G . G G G G G .    
574	T T T T T T T T . T T T T T .    
575	A A A A A A A A . A A A A A .    
576	G G G G G G G G . G G G G G .    
577	A A A A A A A A . A A A A A .    
578	T T T T T T T T . T T T T T .    
579	T T T T T T T T . T T T T T .    
580	T T T T T T T T . T T T T T .    
581	T T T T T T T T . T T T T T .    
582	C C C C C C C C . C C C C C .    
583	C C C C C C C C . C C C C C .    
584	T T T T T T T T . T T T T T .    
585	G G G G G G G G . G G G G G .    
586	T T T T T T T T . T T T T T .    
587	C C C C C C C C . C C C C C .    
588	A A A A A A A A . A A A A A .    
589	T T T T T T T T . T T T T T .    
590	C C C C C C C C . C C C C C .    
591	. C C C C C C C . C C C C C .    
592	. T T T T T T T . T T T T T .    
593	. C C C C C C C . C C C C C .    
594	. T T T T T T T . T T T T T .    
595	. T T T T T T T . T T T T T .    
596	. T T T T T T T . T T T T T .    
597	. G G G G G G G . G G G G G .    
598	. A A A A A A A . A A A A A .    
599	. G G G G G G G . G G G G G .    
600	. G G G G G G G . G G G G G .    
601	. T T T T T T T . T T T T T .    
602	. G G G G G G G . G G G G G .    
603	. T T T T T T T . T T T T T .    
604	. G G G G G G G . G G G G G .    
605	. A A A A A A A . A A A A A .    
606	. A A A A A A A . A A A A A .    
607	. T T T T T T T . T T T T T .    
608	. C C C C C C C . C C C C C .    
609	. C C C C C C C . C C C C C .    
610	. . C C C C C C . C C C C C .    
611	. . A A A A A A . A A A A A .    
612	. . C C C C C C . C C C C C .    
613	. . T T T T T T . T T T T T .    
614	. . G G G G G G . G G G G G .    
615	. . G G G G G G . G G G G G .    
616	. . G G G G G G . G G G G G .    
617	. . G G G G G G . G G G G G .    
618	. . G G G G G G . G G G G G .    
619	. . G G G G G G . G G G G G .    
620	. . G G G G G G . G G G G G .    
621	. . G G G G G G . G G G G G .    
622	. . G G G G G G . G G G G G .    
623	. . G G G G G G . G G G G G .    
624	. . G G G G G G . G G G G G .    
625	. . G G - G G G . G G G G - .    
626	. . G G - G G G . G G G G - .    
627	. . - . - - G G . G G G - - .  5	 indel  
628	. . T . T T T T . G T T T T .    
629	. . G . G G G G . G G G G G .    
630	. . . . G G G G . G G G G G .    
631	. . . . - - - - . G - - - - .    
632	. . . . T T T T . T T T T T .    
633	. . . . C C C C . C C C C C .    
634	. . . . C C C C . C C C C C .    
635	. . . . A A A A . A A A A A .    
636	. . . . A A A A . A A A A A .    
637	. . . . C C C C . C C C C C .    
638	. . . . G G G G . G G G G G .    
639	. . . . T T T T . T T T T T .    
640	. . . . G G G G . G G G G G .    
641	. . . . G G G G . G G G G G .    
642	. . . . A A A A . A A A A A .    
643	. . . . A A A A . A A A A A .    
644	. . . . A A A A . A A A A A .    
645	. . . . A A A A . A A A A A .    
646	. . . . - - - T . T - - T T .    
647	. . . . G G G G . G G G G G .    
648	. . . . A A A A . A A A A A .    
649	. . . . C C C C . C C C C C .    
650	. . . . A A A A . A A A A A .    
651	. . . . C C C C . C C C C C .    
652	. . . . A A A A . A A A A A .    
653	. . . . T T T T . T T T T T .    
654	. . . . G G G G . G G G G G .    
655	. . . . T T T T . T T T T T .    
656	. . . . C C C C . C C C C C .    
657	. . . . A A A A . A A A A A .    
658	. . . . G G G G . G G G G G .    
659	. . . . T T T T . T T T T T .    
660	. . . . C C C C . C C C C C .    
661	. . . . T T T T . T T T T T .    
662	. . . . G G G G . G G G G G .    
663	. . . . A A A A . A A A A A .    
664	. . . . C C C C . C C C C C .    
665	. . . . C C C C . C C C C C .    
666	. . . . A A A A . A A A A A .    
667	. . . . T T T T . T T T T T .    
668	. . . . C C C C . C C C C C .    
669	. . . . A A A A . A A A A A .    
670	. . . . C C C C . C C C C C .    
671	. . . . T T T T . T T T T T .    
672	. . . . A A A A . A A A A A .    
673	. . . . C C C C . C C C C C .    
674	. . . . A A A A . A A A A A .    
675	. . . . A A A A . A A A A A .    
676	. . . . C C C C . C C C C C .    
677	. . . . C C C C . C C C C C .    
678	. . . . G G G G . G G G G G .    
679	. . . . C C C C . C C C C C .    
680	. . . . T T T T . T T T T T .    
681	. . . . C C C C . C C C C C .    
682	. . . . C C C C . C C C C C .    
683	. . . . A A A A . A A A A A .    
684	. . . . T T T T . T T T T T .    
685	. . . . C C C C . C C C C C .    
686	. . . . C C C C . C C C C C .    
687	. . . . T T T T . T T T T T .    
688	. . . . G G G G . G G G G G .    
689	. . . . A A A A . A A A A A .    
690	. . . . A A A A . A A A A A .    
691	. . . . C C C C . C C C C C .    
692	. . . . G G G G . G G G G G .    
693	. . . . C C C C . C C C C C .    
694	. . . . T T T T . T T T T T .    
695	. . . . A A A A . A A A A A .    
696	. . . . G G G G . G G G G G .    
697	. . . . T T T T . T T T T T .    
698	. . . . G G G G . G G G G G .    
699	. . . . T T T T T T T T T T .    
700	. . . . T T T T T T T T T T .    
701	. . . . C C C C C C C C C C .    
702	. . . . C C C C C C C C C C .    
703	. . . . T T T T T T T T T T .    
704	. . . . C C C C C C C C C C .    
705	. . . . T T T T T T T T T T .    
706	. . . . G G G G G G G G G G .    
707	. . . . A A A A A A A A A A .    
708	. . . . G G G G G G G G G G .    
709	. . . . A A A A A A A A A A .    
710	. . . . A A A A A A A A A A .    
711	. . . . A A A A A A A A A A .    
712	. . . . A A A A A A A A A A .    
713	. . . . T T T T T T T T T T .    
714	. . . . G G G G G G G G G G .    
715	. . . . G G G G G G G G G G .    
716	. . . . G G G G G G G G G G .    
717	. . . . T T T T T T T T T T .    
718	. . . . G G G G G G G G G G .    
719	. . . . G G G G G G G G G G .    
720	. . . . G G G G G G G G G G .    
721	. . . . A A A A A A A A A A .    
722	. . . . T T T T T T T T T T .    
723	. . . . G G G G G G G G G G .    
724	. . . . T T T T T T T T T T .    
725	. . . . C C C C C C C C C C .    
726	. . . . T T T T T T T T T T .    
727	. . . . G G G G G G G G G G .    
728	. . . . A A A A A A A A A A .    
729	. . . . C C C C C C C C C C .    
730	. . . . T T T T T T T T T T .    
731	. . . . A A A A A A A A A A .    
732	. . . . C C C C C C C C C C .    
733	. . . . A A A A A A A A A A .    
734	. . . . C C C C C C C C C C .    
735	. . . . T T T T T T T T T T .    
736	. . . . C C C C C C C C C C .    
737	. . . . A A A A A A A A A A .    
738	. . . . G G G G G G G G G G .    
739	. . . . A A A A A A A A A A .    
740	. . . . A A A A A A A A A A .    
741	. . . . G G G G G G G G G G .    
742	. . . . C C C C C C C C C C .    
743	. . . . T T T T T T T T T T .    
744	. . . . G G G G G G G G G G .    
745	. . . . T T T T T T T T T T .    
746	. . . . G G G G G G G G G G .    
747	. . . . G G G G G G G G G G .    
748	. . . . A A A A A A A A A A .    
749	. . . . C C C C C C C C C C .    
750	. . . . G G G G G G G G G G .    
751	. . . . G G G G G G G G G G .    
752	. . . . A A A A A A A A A A .    
753	. . . . C C C C C C C C C C .    
754	. . . . C C C C C C C C C C .    
755	. . . . A A A A A A A A A A .    
756	. . . . C C C C C C C C C C C    
757	. . . . C C C C C C C C C C C    
758	. . . . A A A A A A A A A A A    
759	. . . . G G G G G G G G G G G    
760	. . . . A A A A A A A A A A A    
761	. . . . G G G G G G G G G G G    
762	. . . . C C C C C C C C C C C    
763	. . . . T T T T T T T T T T T    
764	. . . . A A A A A A A A A A A    
765	. . . . T T T T T T T T T T T    
766	. . . . G G G G G G G G G G G    
767	. . . . T T T T T T T T T T T    
768	. . . . T T T T T T T T T T T    
769	. . . . G G G G G G G G G G G    
770	. . . . C C C C C C C C C C C    
771	. . . . A A A A A A A A A A A    
772	. . . . G G G G G G G G G G G    
773	. . . . A A A A A A A A A A A    
774	. . . . C C C C C C C C C C C    
775	. . . . A A A A A A A A A A A    
776	. . . . A A A A A A A A A A A    
777	. . . . A A A A A A A A A A A    
778	. . . . A A A A A A A A A A A    
779	. . . . A A A A A A A A A A A    
780	. . . . G G G G G G G G G G G    
781	. . . . A A A A A A A A A A A    
782	. . . . A A A A A A A A A A A    
783	. . . . A A A A A A A A A A A    
784	. . . . G G G G G G G G G G G    
785	. . . . G G G G G G G G G G G    
786	. . . . G G G G G G G G G G G    
787	. . . . G G G G G G G G G G G    
788	. . . . A A A A A A A A A A A    
789	. . . . G G G G G G G G G G G    
790	. . . . G G G G G G G G G G G    
791	. . . . A A A A A A A A A A A    
792	. . . . G G G G G G G G G G G    
793	. . . . G G G G G G G G G G G    
794	. . . . A A A A A A A A A A A    
795	. . . . G G G G G G G G G G G    
796	. . . . T T T T T T T T T T T    
797	. . . . A A A A A A A A A A A    
798	. . . . G G G G G G G G G G G    
799	. . . . A A A A A A A A A A A    
800	. . . . T T T T T T T T T T T    
801	. . . . A A A A A A A A A A A    
802	. . . . G G G G G G G G G G G    
803	. . . . A A A A A A A A A A A    
804	. . . . G G G G G G G G G G G    
805	. . . . T T T T T T T T T T T    
806	. . . . T T T T T T T T T T T    
807	. . . . A A A A A A A A A A A    
808	. . . . A A A A A A A A A A A    
809	. . . . A A A A A A A A A A A    
810	. . . . C C C C C C C C C C C    
811	. . . . G G G G G G G G G G G    
812	. . . . T T T T T T T T T T T    
813	. . . . T T T T T T T T T T T    
814	. . . . C C C C C C C C C C C    
815	. . . . T T T T T T T T T T T    
816	. . . . G G G G G G G G G G G    
817	. . . . C C C C C C C C C C C    
818	. . . . C C C C C C C C C C C    
819	. . . . T T T T T T T T T T T    
820	. . . . T T T T T T T T T T T    
821	. . . . C C C C C C C C C C C    
822	. . . . T T T T T T T T T T T    
823	. . . . G G G G G G G G G G G    
824	. . . . T T T T T T T T T T T    
825	. . . . G G G G G G G G G G G    
826	. . . . A A A A A A A A A A A    
827	. . . . T T T T T T T T T T T    
828	. . . . G G G G G G G G G G G    
829	. . . . T T T T T T T T T T T    
830	. . . . A A A A A A A A A A A    
831	. . . . G G G G G G G G G G G    
832	. . . . A A A A A A A A A A A    
833	. . . . G G G G G G G G G G G    
834	. . . . A A A A A A A A A A A    
835	. . . . A A A A A A A A A A A    
836	. . . . G G G G G G G G G G G    
837	. . . . T T T T T T T T T T T    
838	. . . . G G G G G G G G G G G    
839	. . . . T T T T T T T T T T T    
840	. . . . G G G G G G G G G G G    
841	. . . . T T T T T T T T T T T    
842	. . . . G G G G G G G G G G G    
843	. . . . T T T T T T T T T T T    
844	. . . . G G G G G G G G G G G    
845	. . . . T T T T T T T T T T T    
846	. . . . G G G G G G G G G G G    
847	. . . . - - - T T T - - T T T  5	 indel  
848	. . . . - - - G G G - - G G G  5	 indel  
849	. . . . A A A A A A A A A A A    
850	. . . . A A A A A A A A A A A    
851	. . . . A A A A A A A A A A A    
852	. . . . G G G G G G G G G G G    
853	. . . . A A A A A A A A A A A    
854	. . . . A A A A A A A A A A A    
855	. . . . A A A A A A A A A A A    
856	. . . . G G G G G G G G G G G    
857	. . . . G G G G G G G G G G G    
858	. . . . T T T T T T T T T T T    
859	. . . . A A A A A A A A A A A    
860	. . . . G G G G G G G G G G G    
861	. . . . C C C C C C C C C C C    
862	. . . . A A A A A A A A A A A    
863	. . . . A A A A A A A A A A A    
864	. . . . G G G G G G G G G G G    
865	. . . . A A A A A A A A A A A    
866	. . . . T T T T T T T T T T T    
867	. . . . C C C C C C C C C C C    
868	. . . . A A A A A A A A A A A    
869	. . . . G G G G G G G G G G G    
870	. . . . G G G G G G G G G G G    
871	. . . . G G G G G G G G G G G    
872	. . . . G G G G G G G G G G G    
873	. . . . A A A A A A A A A A A    
874	. . . . A A A A A A A A A A A    
875	. . . . G G G G G G G G G G G    
876	. . . . G G G G G G G G G G G    
877	. . . . T T T T T T T T T T T    
878	. . . . T T T T T T T T T T T    
879	. . . . T T T T T T T T T T T    
880	. . . . A A A A A A A A A A A    
881	. . . . G G G G G G G G G G G    
882	. . . . G G G G G G G G G G G    
883	. . . . A A A A A A A A A A A    
884	. . . . A A A A A A A A A A A    
885	. . . . T T T T T T T T T T T    
886	. . . . T T T T T T T T T T T    
887	. . . . C C C C C C C C C C C    
888	. . . . C C C C C C C C C C C    
889	. . . . C C C C C C C C C C C    
890	. . . . G G G G G G G G G G G    
891	. . . . C C C C C C C C C C C    
892	. . . . T T T T T T T T T T T    
893	. . . . G G G G G G G G G G G    
894	. . . . T T T T T T T T T T T    
895	. . . . A A A A A A A A A A A    
896	. . . . C C C C C C C C C C C    
897	. . . . T T T T T T T T T T T    
898	. . . . G G G G G G G G G G G    
899	. . . . T T T T T T T T T T T    
900	. . . . A A A A A A A A A A A    
901	. . . . T T T T T T T T T T T    
902	. . . . A A A A A A A A A A A    
903	. . . . T T T T T T T T T T T    
904	. . . . G G G G G G G G G G G    
905	. . . . T T T T T T T T T T T    
906	. . . . T T T T T T T T T T T    
907	. . . . G G G G G G G G G G G    
908	. . . . A A A A A A A A A A C    
909	. . . . C C C C C C C C C C C    
910	. . . . C C C C C C C C C C C    
911	. . . . A A A A A A A A A A A    
912	. . . . T T T T T T T T T T T    
913	. . . . T T T T T T T T T T T    
914	. . . . T T T T T T T T T T T    
915	. . . . T T T T T T T T T T T    
916	. . . . C C C C C C C C C C C    
917	. . . . A A A A A A A A A A A    
918	. . . . T T T T T T T T T T T    
919	. . . . G G G G G G G G G G G    
920	. . . . T T T T T T T T T T T    
921	. . . . T T T T T T T T T T T    
922	. . . . G G G G G G G G G G G    
923	. . . . T T T T T T T T T T T    
924	. . . . A A A A A A A A A A A    
925	. . . . A A A A A A A A A A A    
926	. . . . T T T T T T T T T T T    
927	. . . . G G G G G G G G G G G    
928	. . . . T T T T T T T T T T T    
929	. . . . T T T T T T T T T T T    
930	. . . . A A A A A A A A A A A    
931	. . . . T T T T T T T T T T T    
932	. . . . C C C C C C C C C C C    
933	. . . . A A A A A A A A A A A    
934	. . . . T T T T T T T T T T T    
935	. . . . A A A A A A A A A A A    
936	. . . . T T T T T T T T T T T    
937	. . . . C C C C C C C C C C C    
938	. . . . T T T T T T T T T T T    
939	. . . . A A A A A A A A A A A    
940	. . . . A A A A A A A A A A A    
941	. . . . G G G G G G G G G G G    
942	. . . . T T T T T T T T T T T    
943	. . . . C C C C C C C C C C C    
944	. . . . T T T T T T T T T T T    
945	. . . . G G G G G G G G G G G    
946	. . . . C C C C C C C C C C C    
947	. . . . C C C C C C C C C C C    
948	. . . . T T T T T T T T T T T    
949	. . . . C C C C C C C C C C C    
950	. . . . T T T T T T T T T T T    
951	. . . . A A A A A A A A A A A    
952	. . . . T T T T T T T T T T T    
953	. . . . G G G G G G G G G G G    
954	. . . . A A A A A A A A A A A    
955	. . . . T T T T T T T T T T T    
956	. . . . T T T T T T T T T T T    
957	. . . . G G G G G G G G G G G    
958	. . . . G G G G G G G G G G G    
959	. . . . A A A A A A A A A A A    
960	. . . . G G G G G G G G G G G    
961	. . . . G G G G G G G G G G G    
962	. . . . C C C C C C C C C C C    
963	. . . . C C C C C C C C C C C    
964	. . . . A A A A A A A A A A A    
965	. . . . G G G G G G G G G G G    
966	. . . . A A A A A A A A A A A    
967	. . . . C C C C C C C C C C C    
968	. . . . C C C C C C C C C C C    
969	. . . . T T T T T T T T T T T    
970	. . . . G G G G G G G G G G G    
971	. . . . C C C C C C C C C C C    
972	. . . . A A A A A A A A A A A    
973	. . . . C C C C C C C C C C C    
974	. . . . T T T T T T T T T T T    
975	. . . . A A A A A A A A A A A    
976	. . . . G G G G G G G G G G G    
977	. . . . T T T T T T T T T T T    
978	. . . . C C C C C C C C C C C    
979	. . . . A A A A A A A A A A A    
980	. . . . A A A A A A A A A A A    
981	. . . . C C C C C C C C C C C    
982	. . . . T T T T T T T T T T T    
983	. . . . T T T T T T T T T T T    
984	. . . . T T T T T T T T T T T    
985	. . . . T T T T T T T T T T T    
986	. . . . T T T T T T T T T T T    
987	. . . . T T T T T T T T T T T    
988	. . . . A A A A A A A A A A A    
989	. . . . A A A A A A A A A A A    
990	. . . . T T T T T T T T T T T    
991	. . . . G G G G G G G G G G G    
992	. . . . T T T T T T T T T T T    
993	. . . . A A A A A A A A A A A    
994	. . . . A A A A A A A A A A A    
995	. . . . A A A A A A A A A A A    
996	. . . . A A A A A A A A A A A    
997	. . . . A A A A A A A A A A A    
998	. . . . A A A A A A A A A A A    
999	. . . . A A A A - A A A A A A    
1000	. . . . - - A - - - - - - - -    
1001	. . . . T T T T T T T T T T T    
1002	. . . . A A A A A A A A A A A    
1003	. . . . A A A A A A A A A A A    
1004	. . . . T T T T T T T T T T T    
1005	. . . . A A A A A A A A A A A    
1006	. . . . A A A A A A A A A A A    
1007	. . . . T T T T T T T T T T T    
1008	. . . . A A A A A A A A A A A    
1009	. . . . A A A A A A A A A A A    
1010	. . . . T T T T T T T T T T T    
1011	. . . . A A A A A A A A A A A    
1012	. . . . C C C C C C C C C C C    
1013	. . . . A A A A A A A A A A A    
1014	. . . . A A A A A A A A A A A    
1015	. . . . A A A A A A A A A A A    
1016	. . . . A A A A A A A A A A A    
1017	. . . . G G G G G G G G G G G    
1018	. . . . C C C C C C C C C C C    
1019	. . . . C T T T T T T T T T T    
1020	. . . . . G G G G G G G G G G    
1021	. . . . . T T T T T T T T T T    
1022	. . . . . C C C C C C C C C C    
1023	. . . . . T T T T T T T T T T    
1024	. . . . . G G G G G G G G G G    
1025	. . . . . A A A A A A A A A A    
1026	. . . . . G G G G G G G G G G    
1027	. . . . . T T T T T T T T T T    
1028	. . . . . T T T T T T T T T T    
1029	. . . . . G G G G G G G G G G    
1030	. . . . . G G G G G G G G G G    
1031	. . . . . A A A A A A A A A A    
1032	. . . . . A A A A A A A A A A    
1033	. . . . . A A A A A A A A A A    
1034	. . . . . T T T T T T T T T T    
1035	. . . . . A A A A A A A A A A    
1036	. . . . . C C C C C C C C C C    
1037	. . . . . T T T T T T T T T T    
1038	. . . . . T T T T T T T T T T    
1039	. . . . . G G G G G G G G G G    
1040	. . . . . A A A A A A A A A A    
1041	. . . . . A A A A A A A A A A    
1042	. . . . . T T T T T T T T T T    
1043	. . . . . A A A A A A A A A A    
1044	. . . . . A A A A A A A A A A    
1045	. . . . . A A A A A A A A A A    
1046	. . . . . T T T T T T T T T T    
1047	. . . . . G G G G G G G G G G    
1048	. . . . . G G G G G G G G G G    
1049	. . . . . T T T T T T T T T T    
1050	. . . . . T T T T T T T T T T    
1051	. . . . . T T T T T T T T T T    
1052	. . . . . A A A A A A A A A A    
1053	. . . . . C C C C C C C C C C    
1054	. . . . . C C C C C C C C C C    
1055	. . . . . A A A A A A A A A A    
1056	. . . . . G G G G G G G G G G    
1057	. . . . . G G G G G G G G G G    
1058	. . . . . C C C C C C C C C C    
1059	. . . . . T T T C T T T T T T    
1060	. . . . . G G G . G G G G G G    
1061	. . . . . A A A . A A A A A A    
1062	. . . . . A A A . A A A A A A    
1063	. . . . . A A A . A A A A A A    
1064	. . . . . G G G . G G G G G G    
1065	. . . . . A A A . A A . A A C    
1066	. . . . . C C C . C C . C C .    
1067	. . . . . . C T . . T . T T .    
1068	. . . . . . . . . . . . A . .    
1069	. . . . . . . . . . . . T . .
```
